# Narrative reconstruction and embodiment in the Desert Journey program for young adult cancer survivors

**DOI:** 10.1007/s00520-026-11021-1

**Published:** 2026-07-20

**Authors:** Inbar Levkovich, Yaira Hamama-Raz, Liat Hamama, Shira Kuperman, Michal Labes

**Affiliations:** 1 Faculty of Education, Tel Hai University of Kiryat Shmona in the Galilee, Kiryat Shmona, Israel; 2 School of Social Work, Ariel University, Ariel, Israel; 3 School of Social Work, Tel Aviv University, Tel Aviv, Israel; 4 Stop Cancer Organization, Tel Aviv, Israel; 5 Faculty of Criminology, Bar-Ilan University, Ramat Gan, Israel

**Keywords:** Cancer survivors, Narrative approach, Nature-based intervention, Outdoor interventions

## Abstract

**Purpose:**

Young adults diagnosed with cancer face complex challenges related to identity, body perception, and psychosocial adjustment during survivorship. Nature-based rehabilitation programs have been proposed as potential supportive interventions, yet little is known about how participants construct meaning from these experiences. This study explored how young adult cancer survivors narrated the impact of participation in the Desert Journey program, a multi-day desert trek for young adults coping with cancer.

**Methods:**

In-depth semi-structured interviews were conducted with 21 young adult cancer survivors after participating in the program. Data were analyzed through a dual analytic strategy combining narrative analysis to reconstruct individual accounts as evolving plots and cross-narrative thematic analysis to identify shared patterns of change.

**Results:**

Three interconnected themes emerged: (1) the body that tells a story—a shift from experiencing the body as fragile and medically controlled to perceiving it as resilient and capable; (2) rewriting identity—movement from a survival-focused self-definition toward renewed authenticity and belonging; (3) the day after—varying experiences of integrating journey insights into everyday life, with some participants sustaining personal growth and others reporting challenges maintaining changes without continued support.

**Conclusion:**

Nature-based group programs may provide an important context for psychosocial recovery among young adult cancer survivors. The findings highlight the potential value of such interventions within supportive cancer care and emphasize the importance of ongoing psychosocial and peer support to sustain their benefits.

**Supplementary Information:**

The online version contains supplementary material available at 10.1007/s00520-026-11021-1.

## Introduction

Young adult cancer survivors face a distinctive convergence of developmental disruption and existential threat stemming from the diagnosis, medical treatments, and persistent side effects, all of which may worsen their quality of life [1]. A recent systematic mixed-methods review [2] found needs across medical, psychological, fertility/sexuality, healthcare system, social life, support, and daily life domains were reported in more than half of the studies on this population. Young adulthood is typically characterized by identity exploration, intimacy formation, educational and career consolidation, and growing independence [3], but a cancer diagnosis disrupts the life story and reshapes bodies, social roles, and future imaginaries [4]. Beyond the acute medical crisis, survivorship often entails ongoing negotiations with uncertainty, altered embodiment, fractured self-narratives, and challenges of reintegration into social and occupational life [4–7]. While survivorship research has documented both psychological distress [2] and post-traumatic growth [8] and shown the importance of peer support to young adults [9], we know less about how survivors make meaning of transitional, non-clinical experiences designed to catalyze recovery and reorientation.

This study examined how young adult cancer survivors narrated their experiences in the Desert Journey program, a 3-day group trek operated by Stop Cancer (*Halasartan* in Hebrew), a nonprofit organization in Israel that provides a unique social support network for young adult cancer patients. The program combines preparatory group sessions with a multi-day desert trek to create a structured yet flexible environment for reflection, connection, and rebuilding life following cancer treatment. Participants share their stories, support one another, and engage in self-exploration and meaning-making in a non-medicalized context.

The program includes preparatory group meetings followed by a 3-day immersive trek in the desert. The preparatory stage is intended to create a safe group climate, introduce participants to the structure of the journey, and support initial trust among group members and facilitators. During the trek, participants take part in graded walking routes, facilitated group conversations, reflective storytelling exercises, symbolic activities, shared camp routines, meals, and periods of individual quiet time in the natural environment. These components are designed to combine embodied activity, peer interaction, and guided reflection, thereby allowing participants to process illness-related experiences in a non-clinical setting and to explore new meanings of recovery, identity, and belonging.

The program is grounded in an integrated framework that links the biopsychosocial model with ecopsychological approaches. The biopsychosocial paradigm conceptualizes health and illness as arising from reciprocal interactions among biological mechanisms, psychological processes (e.g., emotion, cognition, coping), and social contexts (e.g., relationships, culture, socioeconomic conditions) [10]. In rehabilitation settings, this perspective supports coordinated, person-centered care that addresses symptoms and behaviors while modifying environmental supports to enhance functioning and participation [11].

Complementing this approach, ecopsychology and nature-based therapies treat the natural world as an active therapeutic agent, a co-therapist capable of eliciting transformative experiences, strengthening self-awareness and interpersonal attunement, and leveraging restorative qualities through creative, experiential outdoor practices [12–15]. The desert, for example, can be understood as a liminal setting, an in-between space, that is both physically demanding and symbolically rich. Its harsh conditions and vast openness take people to their limits, requiring careful choices about pace, route, and resources, thus strengthening agency. At the same time, the desert’s simplicity and expansiveness support attentional restoration and flow, as in other natural environments, and invite reflection on identity, goals, and future possibilities [16–18]. These features make the desert a context in which individuals can experience empowerment and healing while reimagining what is possible [17].

Nature-based therapeutic interventions for cancer survivors have gained attention as complementary approaches to traditional supportive care [19].

A growing body of literature supports the relevance of nature-based approaches for health and survivorship care. Nature therapy conceptualizes the natural environment not merely as a backdrop for intervention, but as an active component of the therapeutic process that may facilitate reflection, emotional regulation, embodied awareness, and interpersonal connection [12, 20, 21]. Among cancer survivors, nature-based interventions have been shown to address multiple dimensions of recovery, including psychological distress, bodily reconnection, social belonging, and existential or spiritual meaning [22, 23]. Research on forest bathing, or shinrin-yoku, further demonstrates how immersive exposure to natural environments may contribute to stress reduction and emotional restoration [24]. Relevant evidence also comes from True North Treks, an immersive nature-based program for young adults and caregivers affected by cancer, which emphasizes connection with nature, connection with peers, and connection with oneself through mindfulness-based practices [25]. In a single-arm program evaluation, participation in these multi-night wilderness treks was associated with improvements in nature connection, peer connection, self-reflection, anxiety, depression, and sleep disturbance [25]. In addition, research on young adult cancer survivors’ preferences for supportive care indicates that many survivors value group-based interventions, particularly after completing treatment, when feelings of loneliness, uncertainty, and unmet psychosocial needs may become especially salient [26]. Although the Desert Journey takes place in a desert rather than a forest, this broader literature supports the idea that sustained engagement with natural environments can create conditions for recovery-oriented meaning-making. For young adult cancer survivors in particular, group-based and camp-like nature experiences may be especially relevant because they combine peer belonging, distance from medicalized settings, embodied activity, and opportunities for post-cancer identity reconstruction [5, 27].

Desert environments, with their vast landscapes and challenging terrain, offer unique opportunities for physical and psychological transformation. However, research on the experiences of young adult cancer survivors in these environments remains limited.

We examined how young adult cancer survivors narrated their experiences in the Desert Journey program. Specifically, we explored transformations across the before–during–after sequence of the journey, aiming to contribute a nuanced account of embodied meaning-making in a liminal natural setting, offer insights into the narrative mechanisms through which group-based outdoor programs foster agency, belonging, and reorientation, and suggest implications for the design of post-treatment support that extends beyond the clinic into sustained peer and follow-up structures.

## Method

We employed narrative inquiry as the overarching qualitative framework [28] and used narrative analysis to reconstruct participants’ stories as evolving plots with characters, themes, and temporal structures [29]. Analytically, we examined both content and form, including what was emphasized or silenced, recurring or absent words, and the temporal dimensions of before, during, and after to capture the complexity and multiplicity of the desert experience.

### Participants

The study included 21 young adult cancer survivors (ages 29–45; M = 42.14, SD = 4.3; 17 women, 4 men) who had completed cancer treatments and participated in the multi-day Desert Journey program in Israel. The program is available to young adults aged 18–44 who have completed cancer treatment. All participants met the program’s age criteria at the time of enrollment; the higher ages at the time of the interviews reflect the interval of 3–6 months between participation in the program and data collection (Table 1). No baseline psychosocial assessments or standardized functional-status measures were administered as part of the present qualitative study. Participants’ clinical characteristics were documented through self-reported cancer diagnosis and treatment history. Participants did not receive financial or material compensation for taking part in the interviews. The cancer diagnoses are reported in Table 1; some diagnoses were grouped into broader categories to protect confidentiality, given the small number of participants in several diagnostic subgroups. Participants were recruited in collaboration with the nonprofit organization Stop Cancer, which operates Desert Journey. Recruitment was conducted via announcements posted on the organization’s website and social media platforms, as well as through direct contact by program coordinators, who informed eligible survivors of the study. Interested individuals who met the inclusion criteria contacted the research team directly and received detailed information about the study and consent forms. Stop Cancer approached 80 individuals who had participated in Desert Journey during the previous 2 years. Invitations were distributed through alumni communication channels, including designated WhatsApp groups. Individuals who were interested in participating contacted the research team or responded to the invitation and received detailed information about the study before signing informed consent. The final sample included 21 participants. Interviews continued until thematic saturation was reached. Because recruitment was conducted through group-based communication channels, the exact number of individuals who viewed the invitation but chose not to respond could not be determined.

**Table 1:** Sociodemographic and clinical characteristics of participants (*N* = 21)

Characteristic			*n* (%)
Sociodemographic characteristics		Age (years) mean (SD)	42.14 (4.3)
	Gender	Female	17 (80.9)
		Male	4 (19.1)
	Children	Yes	11 (52.4)
		No	10 (47.6)
	Marital status	Married	12 (57.1)
		Single	5 (23.8)
		Divorced	3 (14.3)
		Widowed	1 (4.8)
	Employment status	Employed	14 (66.7)
		Unemployed	7 (33.3)
Clinical characteristics		Year of cancer diagnosis mean (SD)	2014.85 (5.07)
	Cancer type	Breast cancer	10 (47.6)
		Lymphoma	5 (23.8)
		Other cancer types	6 (28.6)
	Therapies received	Chemotherapy	17 (80.9)
		Radiation therapy	13 (61.9)
		Surgery	12 (57.1)

### Data collection

Data were collected in semi-structured, in-depth narrative interviews. The interview guide was flexible and conversational, beginning with a broad narrative opening question: “Tell me the story of your participation in Desert Journey.” Participants were then invited to elaborate through follow-up questions such as, “How would you describe your experiences during the three days in the desert?” and “What has changed for you since the journey ended?”. The questions presented above are examples from a broader semi-structured interview guide rather than the complete set of questions. The guide included open-ended prompts about participants’ reasons for joining the program, expectations and concerns before the journey, meaningful moments during the trek, physical and emotional challenges, coping resources, relationships with other participants, and perceived changes in their understanding of illness, recovery, and daily life after the program. Follow-up questions were used flexibly to encourage participants to elaborate on experiences that were personally significant to them. The full interview guide is provided as Supplementary File 1. Interviews took place between April 2025 and October 2025 via Zoom, allowing participants to join from locations that were comfortable and convenient for them. The interviews were conducted by two female researchers, one holding a PhD and the other a doctoral candidate, both of whom had expertise in qualitative research. Neither interviewer had a prior relationship with the participants or with the Stop Cancer organization, which helped reduce potential interpersonal or organizational bias during data collection. Interviews lasted 60–90 min and explored participants’ experiences before, during, and after the journey. All interviews were conducted in Hebrew and later translated into English for analysis and publication purposes. They were audio-recorded with participants’ explicit consent. A professional native English-speaking translator performed the translation, and a back-translation procedure was used to ensure linguistic and conceptual accuracy. Interviews were conducted until data saturation was reached. The interview transcripts ranged from 3414 to 8299 words, totaling 106,092 words across all transcripts.

### Data analysis

The interviews were analyzed by two experienced researchers involved in the design and execution of the study. The two analysts were female researchers with formal training and experience in qualitative health research, including narrative and thematic approaches. One analyst held a PhD, and the other was a doctoral candidate. Their prior methodological experience enabled them to conduct the narrative reconstruction, identify temporal and thematic patterns across participants’ accounts, and engage in reflexive discussions throughout the analytic process. To minimize the influence of personal assumptions and values, they applied bracketing techniques, critically reflecting on their own perspectives to remain attentive to participants’ narratives [30]. The analytic process combined narrative and thematic approaches. The first stage involved narrative analysis to reconstruct participants’ accounts as evolving plots [28, 29]. Each transcript was read repeatedly to identify turning points, temporal sequences (before, during, after), central characters, and shifts in narrative voice. This stage emphasized the storied nature of participants’ experiences and the ways meaning was produced in the act of telling. The second stage involved cross-narrative thematic analysis [28, 31] to identify recurrent patterns, conflicts, and shared meanings across participants’ stories. The two researchers collaborated closely during the analysis, with differences in interpretation addressed as part of the broader validation process. This dual analytic strategy generated overarching themes, including the transforming body, the rewriting of identity, and the “day after” the journey, while preserving the unique narrative nature of each account.

As a supplementary descriptive step, we conducted a word-frequency analysis using NVivo. This analysis was not intended to replace the narrative and thematic interpretation, but rather to provide an additional overview of recurring emotional language across participants’ accounts. The analysis focused on selected positive and negative emotion-related terms that emerged through repeated reading of the transcripts. Similar word forms were grouped together when they conveyed the same emotional meaning. Because the interviews were conducted in Hebrew and later translated into English, the frequency output was reviewed contextually to ensure that the counted terms reflected the intended emotional meaning within participants’ narratives.

### Trustworthiness

Trustworthiness was ensured through prolonged engagement, member checking, peer debriefing, and the use of reflexive journals throughout the research process. Researchers maintained ongoing reflexivity by writing journals and holding discussions [30]. Dependability was achieved through systematic documentation throughout the research process, including interview transcripts, field notes, and coding records managed using qualitative analysis software, thereby ensuring transparency and reliability. Confirmability was maintained by grounding the findings in participants’ narratives and triangulating interpretations through team-based coding and consensus-building discussions. In reporting, we adhered to the Consolidated Criteria for Reporting Qualitative Research (COREQ) to ensure transparency and rigor.

### Ethical considerations

The study was approved by the institutional ethics committee. All participants provided informed consent. To protect confidentiality, all names used in the manuscript are pseudonyms, and potentially identifying details were removed or generalized where necessary.

## Results

### Narrative analysis

Participants’ narratives described diverse experiences of life after cancer and the meaning they attributed to participation in the Desert Journey program. For many, the journey was not experienced as a single event but as a significant transitional experience that shaped their understanding of their recovery and personal development. Table 2 presents representative quotations from participants, organized by study themes. To protect participants’ privacy, all names appearing alongside quotations are pseudonyms, and identifying details were removed or generalized where necessary.

**Table 2:** Participant quotations organized by theme (pseudonyms)

Theme	Participant	Quotation
Theme 1. The body that tells a story	Arthur (40 years old; testicular cancer)	“I was worried because I always have concerns now. I do not know how I will wake up each morning. On some mornings, I get stuck in the bathroom. I sleep with devices because of my sleep apnea. So, I was afraid of the physical aspect, of the journey, of the walking, that I wouldn’t be able to manage.”
	Sarah (45 years old; breast cancer)	“Throughout the entire routine of treatments, my body felt like it wasn’t mine. I wasn’t allowed to go out in the sun, wasn’t allowed to carry things, and suddenly going out to the desert signified liberation for me, relief, and being normal again.”
	Rachel (29 years old; cervical cancer)	“Five days before we left for the journey, I sprained my ankle and was worried about how I would walk with a sprained ankle. During the journey, I walked with a walking stick and succeeded. Since then, whenever it’s difficult for me to walk, I tell myself, you managed to walk in the desert with a sprained ankle, so you’ll also succeed with this challenge.”
	Liz (43 years old; breast cancer)	“The group experience helped me take the first steps outside. I realized that we all struggle with the same fears, and that gave me the strength to persevere and reach the summit.”
	Lidia (44 years old; breast cancer)	“In the difficult parts, everyone could choose whether to cope with the difficulty or get in the vehicle. There was a point at which I chose to stay. I didn’t feel compelled to see if I could meet the challenge; I preferred to sit, enjoy the view, and listen to my body in all its aspects.”
Theme 2. Rewriting identity	Linda (40 years old; thyroid cancer)	“I would arrive at treatments wearing jeans and heels. The day after chemotherapy, I went on an annual trip with my son, concealing my nausea and vomiting. I did not want people to know that I was sick at all. And suddenly on the journey, things I had neglected inside and didn’t want to deal with at all surfaced and emerged.”
	Anna (38 years old; breast cancer)	“Everything was so slowly removing layers. After settling in, we walked from where we were. And… and it was amazing. They let us wander alone outside. After a long time of not just wandering around outside by myself. You see and do not see that there are people there. This is good because it feels as if something happens to me or I have difficulty, and someone will be there to catch me. But on the other hand, no one is putting me under such a magnifying glass.”
	Rachel (29 years old; cervical cancer)	“I think that having people in my life who understand exactly what I am talking about, that I do not need to explain to them… Because we met in such an exposed and vulnerable place, I think some covenant was formed there, which is very valuable in life.”
Theme 3. The day after	Jacob (43 years old; colon cancer)	*“*During hospitalizations, everyone takes care of you and is with you. And when it is over, you are suddenly alone. On one hand, I was happy that it was over; on the other hand, I did not know where I was returning to. To a job that I did not love? The journey gave me the courage to look for other jobs and try to fulfill myself more.”
	Mia (41 years old; ovarian cancer)	“Following the journey, I became more attentive to what troubles me and what overwhelms me, and I started looking at things through a different lens… It helps me manage my emotions and say to someone, for example, ‘I needed you at a certain moment, and you weren’t there for me,’ and express it so it doesn’t stay on me. It’s important to take these weights off. There are so many things I need to process and to decide with myself what was good for me and what wasn’t, and how I want to see things moving forward… so that next time, if unexpected things happen, I’ll know how to deal with them. I also understand that reality involves even less control than I had thought.”
	Ben (40 years old; lymphoma)	“Today I feel loneliest in the world.”
	Rebecca (31 years; Hodgkin’s lymphoma)	“I don’t know how long it will take me to recover”
	Sarah (38 years old; breast cancer)	“It was very difficult to finish the treatments and hear everyone say, ‘That’s it, it’s behind you now,’ because it doesn’t work like that. The real emotional difficulty began afterward. During treatment I was in survival mode and didn’t have the possibility to deal with my feelings, but afterward I suddenly understood what I had been through. Inside, I felt completely lost, how do you return to life? Even though I was no longer considered sick, I still felt sick. The journey was very meaningful along the way, because hearing others in a similar situation gave me validation and helped me understand that I was not alone.”

**Fig. 1:**
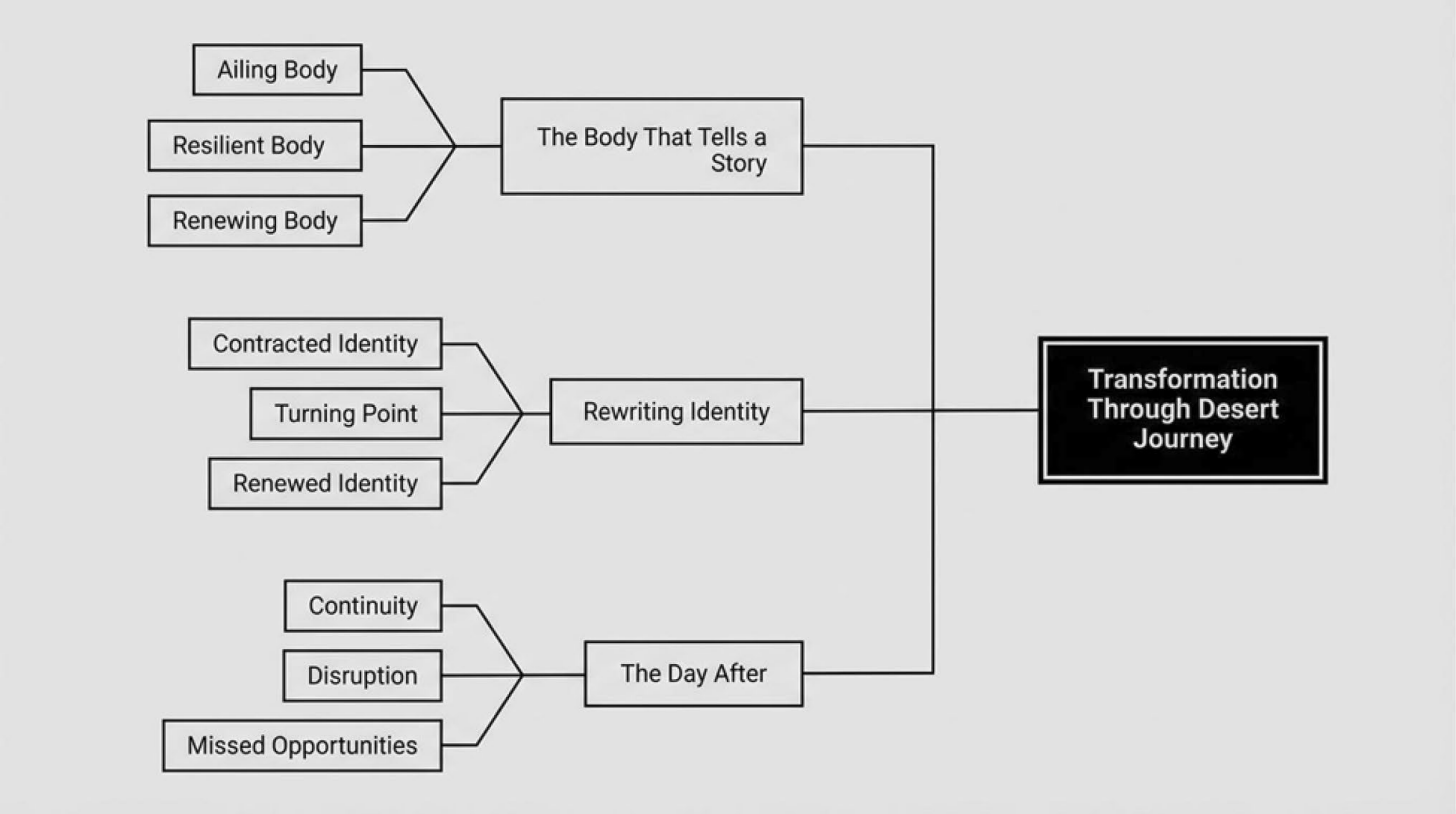
Narrative model of transformation following participation in the Desert Journey program

The narrative analysis focused on four dimensions: time, language, action, and characters—that is, how participants situated their experiences across the journey, expressed them linguistically, described key actions, and constructed the roles of significant others in their stories. The analysis generated three main themes describing participants’ experiences of the desert journey and its perceived impact on their lives (Fig. 1).

#### Theme 1: the body that tells a story

The first theme centered on how participants narrated changes in their relationship with their bodies. Across interviews, the body emerged as a central element in participants’ accounts of cancer and recovery. Their narratives revealed a shift from experiencing the body as fragile, alienated, and medically controlled before the journey to perceiving it as capable, resilient, and worthy of renewed trust thereafter.

Before their journey, many participants said they saw their bodies primarily through the lens of illness and medical treatment. Their narratives often reflected a sense of distance from the body, which was portrayed as fragile, unpredictable, and marked by physical limitations. They frequently used clinical or passive language when referring to their bodily experiences, emphasizing treatments, weakness, fatigue, and loss of control. This initial pre-trek perception of the body was accompanied by uncertainty about their ability to meet the physical demands of the desert. Many participants mentioned apprehension about confronting their bodies in such a challenging environment. At the same time, several said they wanted to reconnect with their bodily sensations and regain a sense of trust in their physical capabilities.

Participants’ narratives began to shift when they talked about physical challenges and movement in the desert landscape. They described themselves as active agents capable of physical endurance and adaptation. Their language reflected this change, emphasizing movement and action through walking, climbing, breathing, and coping. A particularly meaningful turning point often occurred during a demanding ascent in the desert terrain. Reaching the summit was described by several participants as a powerful moment that symbolized the possibility of physical capability beyond what they had previously imagined. In these accounts, the body was no longer described as vulnerable but as a source of strength and accomplishment. Participants also emphasized the importance of the group context in shaping this renewed bodily narrative. Shared physical challenges and mutual encouragement contributed to a sense of collective resilience and reinforced participants’ confidence in their physical abilities. When describing life after the journey, participants portrayed the body less as a site of illness and more as a source of agency, growth, and renewed trust.

#### Theme 2: rewriting identity

The second theme focused on how participants narrated changes in their sense of identity following participation in the Desert Journey program. Across interviews, participants described a shift from an identity defined by illness and survival to a broader sense of self that encompassed belonging, purpose, and engagement with everyday life.

Many participants said that before they went on the journey, their identities were closely tied to the experience of cancer and its aftermath. Their narratives centered on survival, medical follow-up, and ongoing health concerns. They often described feeling confined within a medicalized identity in which being a “cancer survivor” became the dominant lens through which they understood themselves and were perceived by others. Some experienced this identity as restrictive, limiting their ability to reconnect with aspects of life unrelated to illness.

Participation in the desert journey often marked a significant turning point in the identity narratives. The physical and social environment of the journey created a space in which participants could temporarily step outside the routines and roles associated with illness. When they talked about the journey, they began to narrate themselves not only as individuals who had survived cancer but also as people capable of challenge, exploration, and personal development. The desert experience thus served as a transitional context, enabling individuals to reconsider the role of cancer in their broader life stories.

For many participants, the group context of the desert journey played a central role in the emergence of new identity narratives. Through shared experiences and conversations with others who had faced similar challenges, they developed a stronger sense of belonging and mutual understanding. Several participants described channeling their cancer experience into helping or supporting others facing illness, transforming their survival narrative into a sense of purpose and contribution. In these accounts, identity was increasingly framed not only in relation to illness but also in terms of connection, meaning, and engagement with others.

#### Theme 3: the day after the journey

The third theme focused on participants’ experiences after returning from the journey and the challenges involved in integrating the insights gained during the program into everyday life. While many described the journey as a powerful and meaningful experience, their post-journey narratives revealed varying degrees of success in sustaining these changes.

For some participants, returning to daily routines was accompanied by feelings of loneliness and disconnection. The sense of closeness and shared understanding developed during the desert journey often diminished once they returned to their regular environments. Several described a tension between the sense of authenticity and personal insight they experienced during the journey and the social roles and expectations they resumed afterward. This transition sometimes created a feeling of fragmentation, as they struggled to maintain the emotional and personal changes emerging from the desert experience.

Another narrative centered on the perception that the program ended too abruptly. Some participants felt the journey initiated an important process of reflection and change, but the absence of continued support limited how far this change could be sustained. They frequently expressed a desire for opportunities to reconnect with the group or participate in follow-up activities to help them integrate the experience into their ongoing recovery. In these accounts, the need for structured continuation and peer support emerged as an important component in maintaining the program’s benefits.

### Frequency analysis

**Fig. 2:**
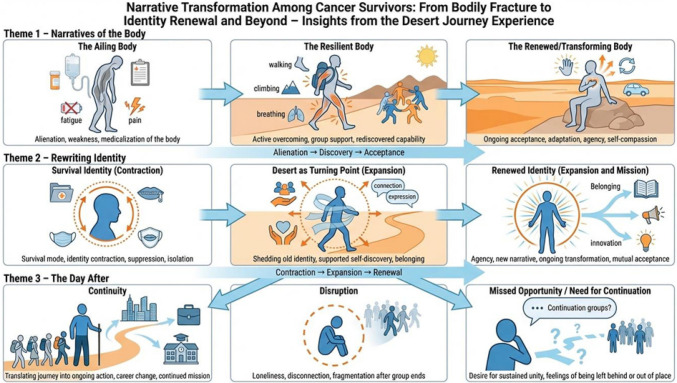
Overview of narrative themes emerging from the Desert Journey experience

Figure 2 presents a frequency analysis of emotion words across 21 narrative interviews. The figure reveals a clear contrast between positive and negative emotional language in participants’ accounts.

**Fig. 3:**
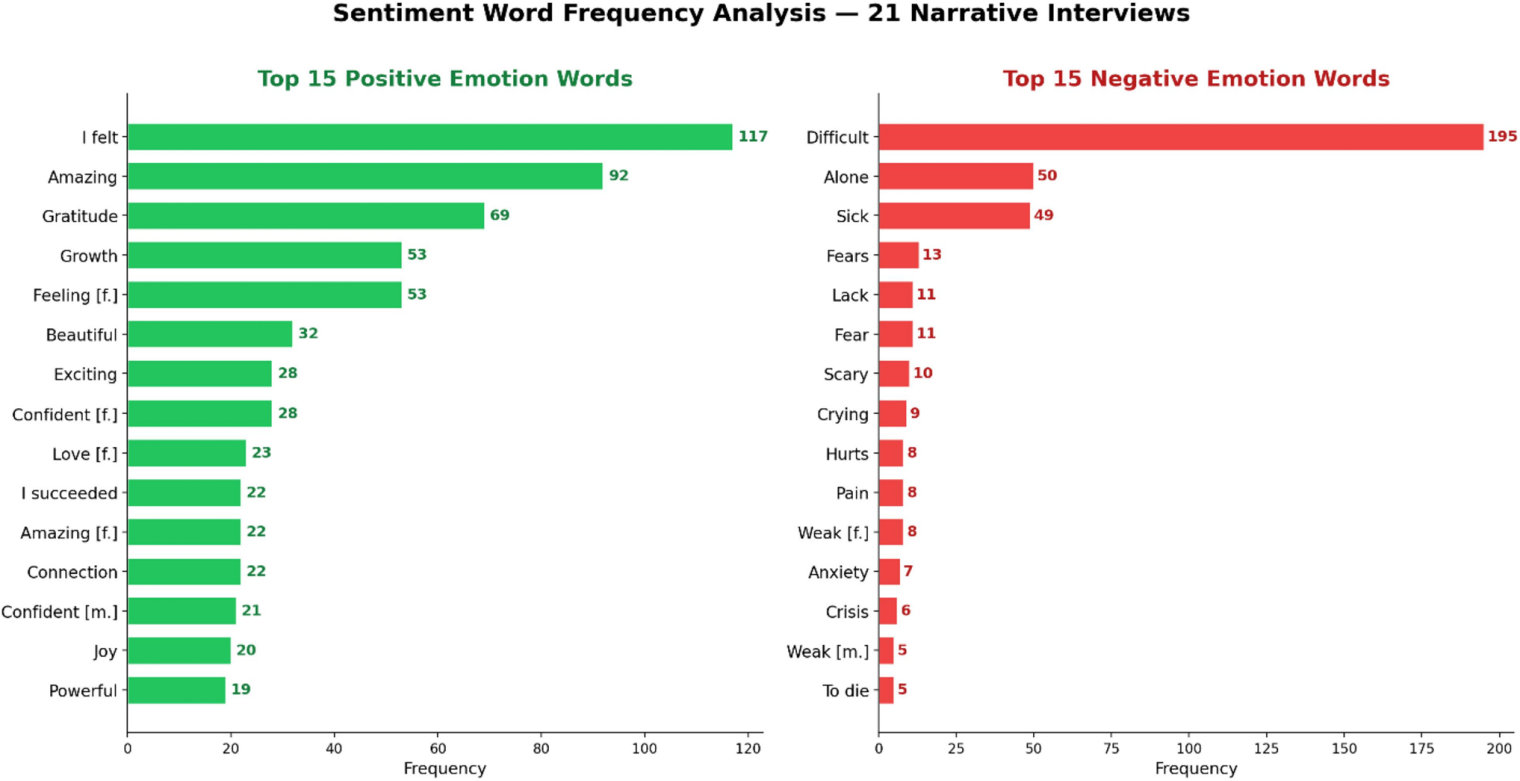
Frequency of positive and negative emotion words across 21 narrative interviews

On the positive side, words such as “I felt,” “amazing,” “gratitude,” and “growth” were especially prominent, suggesting that participants viewed the experience in terms of emotional intensity, appreciation, and personal development. On the negative side, the word difficult stood out with a substantially higher frequency than other negative words, followed by alone and sick, indicating the presence of hardship, loneliness, and emotional or physical distress. This suggests participants’ narratives were not one-dimensional, but combined descriptions of struggle, pain, and challenge with expressions of strength, growth, connection, and meaning. Figure 3 presents the frequency of positive and negative emotion words across the 21 narrative interviews.

In this sense, the emotional discourse reflected in the interviews points to a complex experience that was perceived as both demanding and empowering.

**Fig. 4:**
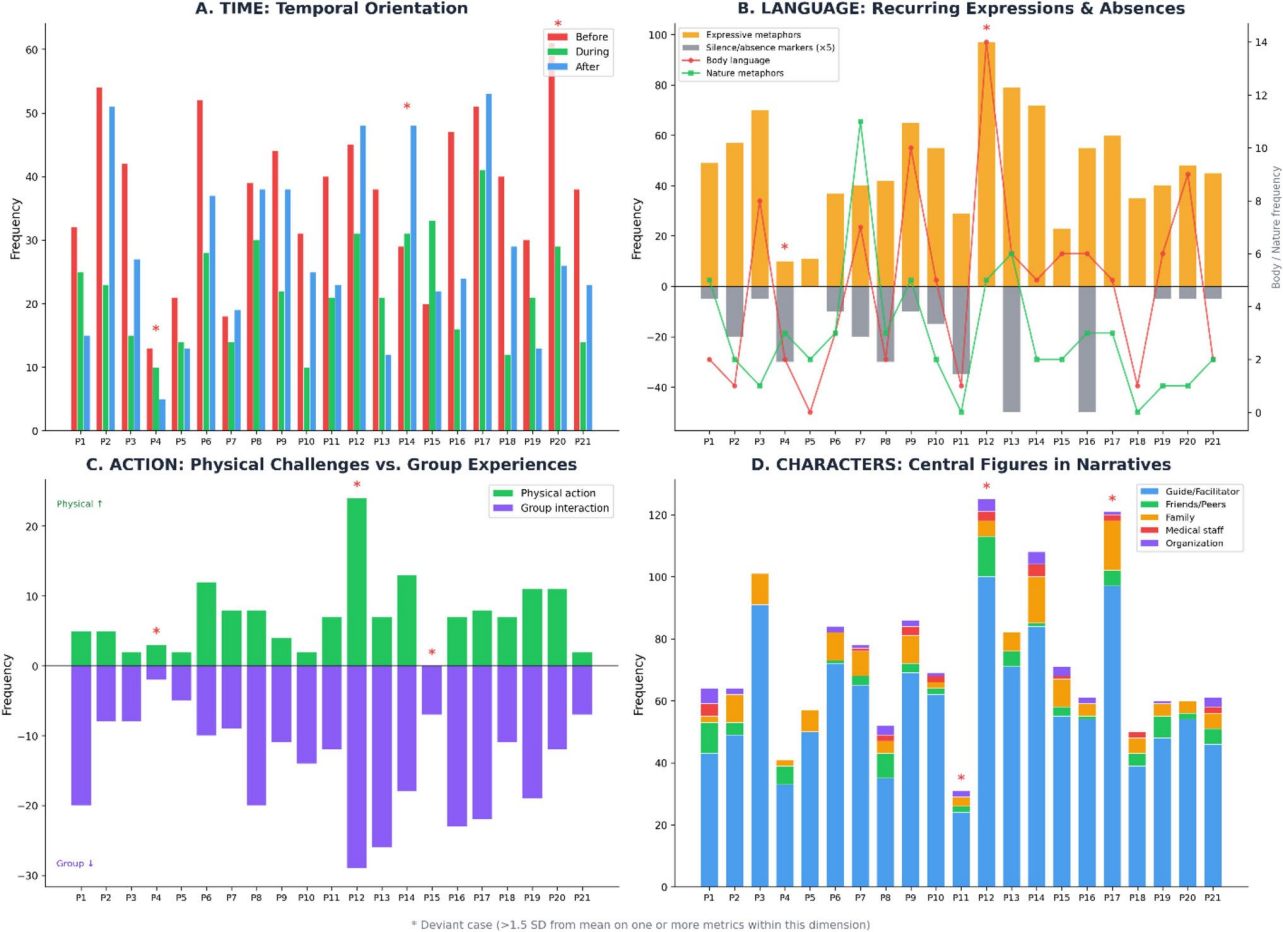
Four analytical dimensions across 21 narrative interviews: time, language, action, and characters

To further examine the narrative structure across the interviews, we analyzed four cross-cutting dimensions: time, language, action, and characters (Fig. 4). References to the period before the journey were most frequent, followed by after and during, suggesting the desert experience served as a lens for reinterpreting illness and recovery. Linguistic patterns included frequent metaphorical, body-related, and nature-based expressions, while action references were primarily physical and group-oriented. The guide emerged as the most central character across narratives, underscoring the relational and supportive structure of the experience.

## Discussion

This study explored the experiences of young adult cancer survivors who participated in a multi-day desert journey. The findings illuminate how nature-based group experiences facilitate post-cancer growth and identity reconstruction. Three interconnected themes emerged from the narrative analysis: the body that tells a story, rewriting identity, and the day after.

The first theme, the body that tells a story, showed how participants’ relationships with their bodies evolved from disconnection and mistrust to renewed connection and acceptance. The transformation reflects an embodied process of meaning-making in which post-treatment embodiment involves not a return to a pre-illness body but the creation of a “new normal,” grounded in the acceptance of change and renewed bodily confidence [32]. In our study, these processes were amplified by shared physical experiences and mutual encouragement within the group, reinforcing collective resilience and belonging. Thus, the body became not only a site of pain and recovery but also a narrative medium through which participants redefined strength, acceptance, and continuity in life after cancer.

The second theme, rewriting identity, captured how participants sought to redefine themselves beyond the boundaries of illness, moving from survival-centered narratives toward a renewed sense of living. The desert journey served as a narrative and experiential turning point, allowing participants to re-author their life stories and reclaim agency over their self-definitions. Previous research has found young cancer survivors often describe identity as a dynamic and ongoing reconstruction, in which past illness remains part of the self but no longer dominates it [33]. Similarly, cancer survivorship involves navigating a complex interplay between vulnerability and strength while striving to regain normalcy and social participation [34]. Identity recovery therefore extends beyond physical healing to include renewed meaning and belonging. We found the desert landscape offered both physical and symbolic distance from the medicalized environment, facilitating a sense of self-continuity and narrative coherence. Other research has suggested the “survivor” label can both empower and constrain individuals, depending on whether it aligns with their evolving sense of self [35]. In our study, the desert journey thus became a lived metaphor for psychological growth, where stories of endurance gave way to narratives of renewed meaning, authenticity, and belonging.

This finding also highlights the broader role of storytelling in recovery after cancer. From a narrative perspective, people make sense of disruptive life events by organizing them into stories that create temporal order, coherence, and meaning [28, 29]. In the present study, the desert provided a symbolic and experiential setting in which participants could retell their illness narratives from a different position: not only as patients or survivors, but as individuals moving through challenge, uncertainty, and renewal. The desert has long been associated with passage, solitude, testing, and transformation; in this study, these symbolic qualities were not merely metaphorical but were embodied through walking, exposure, silence, and shared storytelling. Thus, the desert functioned as a narrative landscape in which participants could transform fragmented illness experiences into more coherent stories of endurance, agency, and belonging.

The third theme, the day after, when the journey ends and insights are revealed, reflected the tension between transformation and continuity in participants’ lives following the desert journey. Their narratives resonate with the framework of post-traumatic growth, which suggests confronting adversity can lead to positive psychological change, deeper relationships, and shifts in life priorities [36, 37]. However, research on survivorship transitions indicates such growth is rarely linear or uniformly sustained [38, 39]. In our study, some participants integrated their desert insights into purposeful life changes, such as mentoring, creative expression, or community involvement, reflecting what has been described as the “action phase” of growth [40]. Others reported diminished momentum, loneliness, or unmet expectations once the group disbanded, echoing findings that growth and distress often coexist and fluctuate over time [41]. The desert journey therefore functioned as a transitional space in which survivors temporarily experienced clarity and empowerment, while the post-journey period revealed the challenges of sustaining transformation without continued support. Participants’ calls for follow-up encounters or peer-led groups indicate post-traumatic growth is not an endpoint but an evolving and relational process sustained through connection, acknowledgment, and opportunities to revisit the meanings discovered during the journey. Importantly, the need for follow-up should be understood as one of the central findings of this study rather than only as a practical recommendation. The intensity of the desert journey created a temporary space of connection, validation, and clarity, yet participants’ narratives showed that the transition back to everyday life could reactivate loneliness, uncertainty, and the difficulty of sustaining change. This pattern is consistent with research on survivorship transitions, which shows that the period after treatment often involves ongoing psychosocial needs and fluctuating experiences of distress and growth [38, 39]. It also aligns with evidence that peer support can contribute to post-traumatic growth among adolescent and young adult cancer survivors [9]. Follow-up meetings or peer-led groups may therefore serve as a bridge between the transformative experience of the journey and the ongoing work of integrating its meanings into daily life.

The desert experience was more than a therapeutic event. It functioned as a transformative space in which illness-related narratives were reinterpreted and integrated into broader life stories. The findings highlight the desert journey as an embodied and socially mediated process of reconstruction, through which participants reconnected with their bodies, reauthored their narratives, and sought ways to sustain these insights beyond the journey. In line with embodiment theory [42, 43], the physical challenges of the desert appeared to restore bodily awareness and agency, allowing participants to experience the body not only as vulnerable, but also as capable and trustworthy. This aligns with previous research showing that contact with nature can help cancer survivors reframe bodily changes and negotiate the tension between vulnerability and vitality [44, 45].

### Implications for practice

The findings have several implications for supportive cancer care. First, oncology and rehabilitation teams may consider referring young adult survivors to structured nature-based group programs as a complement to conventional post-treatment support. Second, program design should incorporate graded physical challenges, facilitated peer interaction, and guided narrative reflection to address bodily, identity, and psychosocial dimensions of recovery simultaneously. Third, follow-up sessions or peer-led support groups should be integrated into program structures to help sustain gains beyond the immediate experience. These elements may be adapted across diverse nature-based settings and survivorship populations.

### Limitations

Several limitations should be acknowledged. First, the study was based on a purposive sample recruited through a single Israeli NGO and included a predominantly female group of participants, which may limit the transferability of the findings to other survivor populations, cultural contexts, and nature-based programs. Second, individuals who declined participation or who did not remain connected to the organization were not included, leaving potential barriers to engagement unexamined. Third, the study did not include baseline psychosocial assessments, objective outcome measures, or a comparison group; therefore, the findings should be interpreted as participants’ perceived experiences rather than evidence of causal effects of the Desert Journey program. In addition, clinical and functional variables, such as cancer stage, treatment-related limitations, coping style, or functional status, were not systematically assessed, limiting our ability to examine how these factors may have shaped participants’ narratives. Fourth, the retrospective interview design may have introduced recall bias, although the interviews allowed participants to reflect meaningfully on the longer-term significance of the journey. Finally, the frequency analysis was used only as a descriptive supplement to the qualitative narrative analysis and should not be interpreted as an independent psycholinguistic assessment. Future studies should incorporate longitudinal designs, baseline and follow-up psychosocial measures, functional assessments, and comparison groups to examine more systematically how nature-based group programs may support young adult cancer survivors over time.

## Conclusion

The findings suggest that nature-based group programs may offer a meaningful supportive context in which young adult cancer survivors can reflect on psychosocial, embodied, and existential aspects of recovery that are not always fully addressed in conventional rehabilitation settings. Rather than demonstrating causal effects, this study highlights how participants perceived the Desert Journey as a space for reconnecting with the body, reworking illness-related narratives, and experiencing peer belonging. Structured reflection, peer interaction, and graded physical challenges may therefore be considered promising components for future supportive-care programs. The findings also underscore the importance of continued psychosocial and peer support after intensive rehabilitation experiences, as follow-up structures may help participants integrate the meanings and insights generated during the program into everyday life.

## Supplementary Information

Below is the link to the electronic supplementary material.

ESM 1 (DOCX 27.2 KB)

## Data Availability

The data supporting the findings of this study are available from the corresponding author upon reasonable request.
