# Supplementary material for: Narrative reconstruction and embodiment in the Desert Journey program for young adult cancer survivors
Source: Support Care Cancer. 2026 Jul 20;34(8):781. doi: 10.1007/s00520-026-11021-1 (PMC13384976; doi:10.1007/s00520-026-11021-1)
Supplement: Supplementary file 1 — (DOCX 27.2 KB) [file 520_2026_11021_MOESM1_ESM.docx]

**Supplementary File 1: Semi-Structured Interview Guide**

| **Interview domain** | **Questions** |
| --- | --- |
| Opening narrative | Tell me the story of your participation in the Desert Journey program. |
| Before the journey | How did you experience the period before the journey? What expectations, concerns, or hopes did you bring with you? |
| The body and physical experience | How did you experience your body before and during the journey? Were there moments in which your perception of your body changed? |
| The desert experience | How would you describe your experience during the three days in the desert? Were there specific moments in the landscape that were meaningful for you? |
| Identity and self-understanding | Did the journey affect how you see yourself after cancer? Did it change how you understand your illness or recovery story? |
| Group and peer relationships | How would you describe your connection with the other participants? Were there moments of support, belonging, distance, or difficulty? |
| Meaning-making and storytelling | Looking back, what meaning do you give to the journey? How would you describe the story of the journey in your life? |
| After the journey | What has changed for you since the journey ended? Which experiences or insights stayed with you? |
| Integration into everyday life | Was it easy or difficult to carry the experience into daily life? What kind of support, if any, was missing after the program ended? |
| Closing question | Is there anything else about the Desert Journey experience that is important for us to understand? |
